# Influence of Exposure to Benzo[a]pyrene on Mice Testicular Germ Cells during Spermatogenesis

**DOI:** 10.1155/2013/387850

**Published:** 2013-12-23

**Authors:** Hueiwang Anna Jeng, Silvina M. Bocca

**Affiliations:** ^1^School of Community and Environmental Health, College of Health Sciences, Old Dominion University, 4608 Hampton Boulevard, Health Sciences Building, Room 3140, Norfolk, VA 23829, USA; ^2^The Jones Institute for Reproductive Medicine, Eastern Virginia Medical School, Norfolk, VA 23829, USA

## Abstract

The objective of this study was to assess the toxicological effect of exposure to benzo(a)pyrene, B[a]P, on germ cells during spermatogenesis. Mice were exposed to B[a]P at 1, 10, 50, and 100 mg/kg/day for 30 days via oral ingestion. Germ cells, including spermatogonia, spermatocytes, pachytene spermatocytes, and round spermatids, were recovered from testes of mice exposed to B[a]P, while mature spermatozoa were isolated from vas deferens. Reproductive organs were collected and weighed. Apoptotic response of germ cells and mature spermatozoa were qualified using the terminal deoxynucleotidyl transferase mediated deoxy-UTP nick end labeling (TUNEL) assay. B[a]P exposure at ≤10 mg/kg/day for 30 days did not significantly alter concentrations of germ cells and mature spermatozoa and apoptotic response in germ cells and mature spermatozoa. Exposure to B[a]P at 50 and 100 mg/kg/day induced testicular atrophy and yielded a significant reduction in the concentrations of spermatogonia, spermatocytes, pachytene spermatocytes, and round spermatid cells as compared with the control. Also, mature spermatozoa experienced decreased concentrations and viability. B[a]P-exposed mice experienced a significant increase in apoptotic germ cells as compared to the control mice. However, the mice dose concentrations were not relevant for comparison to human exposure.

## 1. Introduction

Benzo[a]pyrene (B[a]P), a polycyclic aromatic hydrocarbon compound, is produced by incomplete combustion of organic compounds and high-pressure processes. This compound is commonly present in motor vehicle exhaust, tobacco smoke, grilled, smoked and broiled foods, and emissions from residential and industrial heating sources. Biotransformation of B[a]P occurs as it undergoes initial oxidation by cytochrome P450 enzymes and consequently forms reactive metabolites, for example, B[a]P-9, BaP-7,8-dihydrodiol 9,10-epoxide and quinones. The active intermediates are capable of covalently bonding to DNA [1] and form DNA adducts. Also, they can undergo redox cycling and generate excessive reactive oxygen species, which may alter cell signaling and damage cellular membranes resulting in apoptosis [2].

Previous studies in animal models have demonstrated that direct exposure to B[a]P could cause toxic effects on male reproduction and has been implicated in the pathobiology of adverse reproductive health. B[a]P has been related to decreased spermatozoa quality [3, 4] and germ cell apoptosis after chronic exposure [5]. To date, limited data has appeared available to depict the impact of exposure to B[a]P on spermatozoa quality in relation to testicular germ cells during spermatogenesis. Spermatogenesis represents a complex and dynamic process of proliferation and differentiation of the transformation of spermatogonia into mature spermatozoa in three major stages, the mitotic stage, the meiotic stage, and the maturation stage [6]. Each of these stages represents a key element in the spermatogenic process. Alterations occurring in any of them could lead to the production of abnormal spermatozoa and reduce proliferation of spermatozoa. Thus, the understanding of processes related to spermatogenesis is critical for assessment of male reproductive health.

The objective of this study was to investigate the toxicological impact of B[a]P exposure on testicular germ cells during spermatogenesis by assessing concentrations of and apoptosis in testicular germ cells and mature spermatozoa.

## 2. Materials and Methods

### 2.1. Experimental Protocol

Hsd: ICR (CD1) 10-week-old male mice, weighing 30–40 g, were used (Harlan Laboratories, Inc.). Mice were caged individually with 12 hr light/dark cycles, given rodent chow (Global Rodent Diet number 2018), water *ad libitum*, and enrichment items. All procedures were performed in accordance with protocols approved by the Old Dominion University's Institutional Animal Care and Use Committee. Each mouse was weighed every other day and was examined daily for behavioral and clinical symptoms. Upon arrival, mice were acclimated for two days before B[a]P exposure began. Mice (*n* = 8 per group) were randomly assigned to the exposure group and control group. The exposed mice were gavaged with 1, 10, 50, and 100 mg/kg/day of B[a]P dissolved in 100% corn oil for 30 days. Control animals were only dosed with 20 *μ*L of 100% corn oil. On day 30, mice were anesthetized using intraperitoneal injection of sodium pentobarbital (50 mg/mL) to the surgical plane, confirmed by the toe-pinch method. Reproductive tissues, including testes, seminal vesicles, prostate, epididymis, and vase deferens, were removed and placed on a petri dish and weighed. Vase deferens was used immediately to recover mature spermatozoa. The right testes were placed in 10% formalin for histological preparation. The left testes were used for collection of germ cells and stored in the freezing media-RPMI 1640 and 5% glycerol at −80°C until germ cell collection.

### 2.2. Histological Evaluation of Testes

The testes were fixed with 10% formalin in a 1% phosphate buffer at room temperature for 24–48 hours. Testes were then dehydrated using ethanol and xylene. After dehydration, the testes were embedded in paraffin wax. Then, serial sections (8 *μ*m) were cut from the middle of each testis using a microtome. These sections were placed in a water bath at 47°C and transferred to charged microscope slides. Sections were dried overnight on glass slides at 35°C and deposited in a storage box at room temperature until processed for histology. For staining and morphometric evaluation, sections were deparaffinized and rehydrated using 100%, 95%, and 70% graded ethanol. Slides were stained with hematoxylin and eosin. Images were acquired using an Olympus CX41 microscope with a DP72 camera and CellSens Standard software.

### 2.3. Collection of Germ Cells

Testes were digested in a 50 mL conical tube containing 10 mL D-Hanks solution with 0.5 mg/mL collagenase and 25 *μ*g/mL DNase, then incubated at room temperature for 10 minutes with gentle oscillation. Fetal bovine serum (FBS) was added to halt digestion. Next, the suspension was filtered through a 150 mesh (104 *μ*m) filter to collect the seminiferous tubules. The filtrate was centrifuged at 300 g for 10 minutes to concentrate the interstitial cells. The pellet was suspended in HBSS and stored at −80°C. Cells were pelleted by centrifugation (300 g for 10 minutes), and the supernatant was removed. The cells were then suspended in 0.2% bovine serum albumin (BSA) in RPMI+ by gentle pipetting.

Sedimentation at Unit Gravity was used to separate each type of germ cell, including spermatogonia, pachytene spermatocytes, round spermatids, and elongated spermatids. The procedures were performed according to those developed for murine spermatogenic cells [7, 8] with some modifications. A small STA-PUT chamber and sample loading chamber (Pro Science Glass Co., Ontario, Canada) were prepared by sterilization. The chambers were connected with plastic tubing running through a pump. Both chambers were primed by applying 25 mL RPMI+ through the sample loading chamber using the pump to advance the volume into the STA-PUT chamber. A 10 mL cell suspension was loaded onto a 5 mL cushion of 0.2% BSA in RPMI+ in the sample loading chamber, and then 0.2% BSA in RPMI+ was added at a flow rate of 20 mL/min. The cells were allowed to settle and lie undisturbed for eight hours. The tubing was reversed to allow 10 mL fractions to be drawn from the chamber at a flow rate of 10 mL/min. Each fraction was centrifuged at 900 g for 10 min, and then supernatant was removed. Each fraction was next assessed for cell types, which were identified based on morphology [9]. Large cells with a high nucleocytoplasmic ratio and fine chromatin were considered spermatogonia. Pachytene spermatocytes were identified as cells with an interphase nucleus, and visible cytoplasm. Cells with a small interphase and condensed nucleus were considered as round spermatids. Elongated spermatids were identified based on shape, placement of cytoplasm, condensed nucleus, and the tail. Each type of cell was counted using a hemocytometer and stored at −80°C until apoptosis analysis.

### 2.4. Collection of Mature Spermatozoa and Quality Analysis

A consistent length of each vas deferens, 1.5 cm, was flushed with 0.5% BSA in RPMI+ to recover spermatozoa. Spermatozoa quality, including concentration, viability, motility, and morphology, was assessed. Spermatozoa were counted using a Makler chamber according to manufacturer's instructions. The percentage of motility was determined by counting both motile and immotile spermatozoa. For viability analysis, at least 100 spermatozoa per sample were assessed from an eosin stained preparation. For morphology assessment, two slide smears were prepared for each sample. At least 100 spermatozoa were classified as either normal, abnormal head, or curly tail.

### 2.5. Evaluation of Apoptotic Cells

Apoptotic cells were detected by terminal deoxynucleotidyl transferase mediated deoxy-UTP nick end labeling (TUNEL), using an in situ detection kit (Roche Diagnostic, Manheim, Germany). To detect apoptotic germ cells in seminiferous tubules, testes were fixed in formalin for 24 hours and then embedded in paraffin, and testicular sections were sliced in six *μ*m ribbons on a Leica microtome. Individual sections were mounted on HTC coated slides. The tissue sections were washed with 1% HSA in a 1X phosphorous buffer solution (PBS), permeabilized using 0.1% (v/v) Triton X-100, 0.1% (w/v) sodium citrate in 1X PBS, and incubated at room temperature for 10 minutes. The presence of germ cell apoptosis related to DNA strand breaks was evaluated by terminal deoxynucleotidyl transferase mediated d'UTP nick-end labeling by means of the In Situ Cell Detection Kit with FITC-labeled d'UTP. A TUNEL mixture (Roche) was deployed onto the slide according to the manufacturer's instruction. Slides were incubated for 60 minutes at 32°C, in the dark, with humidity, and were monitored for wetness by adding 1% HSA in 1X PBS as needed. Slides were analyzed immediately using a fluorescent microscope (Nikon Eclipse 80i) with a Cool Snap EZ camera and NIS Elements BR3.2 software. A minimum of 20 fields of view at 1000x magnification were randomly selected for analysis of cells.

To detect apoptotic spermatozoa, 200 *μ*L of spermatozoa at 30 × 10^6^/mL collected from the previous step were washed twice with 1X PBS/1% HSA. Spermatozoa were fixed to the slides, permeabilized with 0.1% Triton X-100 in 0.1% sodium citrate at 4°C for two minutes. A TUNEL mixture was deployed onto spermatozoa according to the manufacturer's instruction. Each test included both positive and negative controls to ensure the performance of the assay. Cells in the positive control were treated with 50 *μ*L of DNase solution, while cells in the negative control did not get treated with the TUNEL mixture. After the cells were incubated one hour at 37°C, the cells were washed twice with 1% HAS in PBS. A minimum of 100 spermatozoa were counted as either positive (green) or negative (absence) at 1000X under oil immersion, using a Nikon Eclipse 80i fluorescent/brightfield microscope with the X-Cite series 120 and FITC filter.

### 2.6. Data Analysis

All data were tested for normality and variance before statistical analysis took place and presented as mean ± standard deviation (SD). Multivariate Analysis of Variances (MANOVA) and LSD *post hoc* tests were utilized to assess statistically significant differences in spermatozoa quality, reproductive organ weight, and apoptotic cells between the exposure group and the control group.

## 3. Results

**Figure 1:**
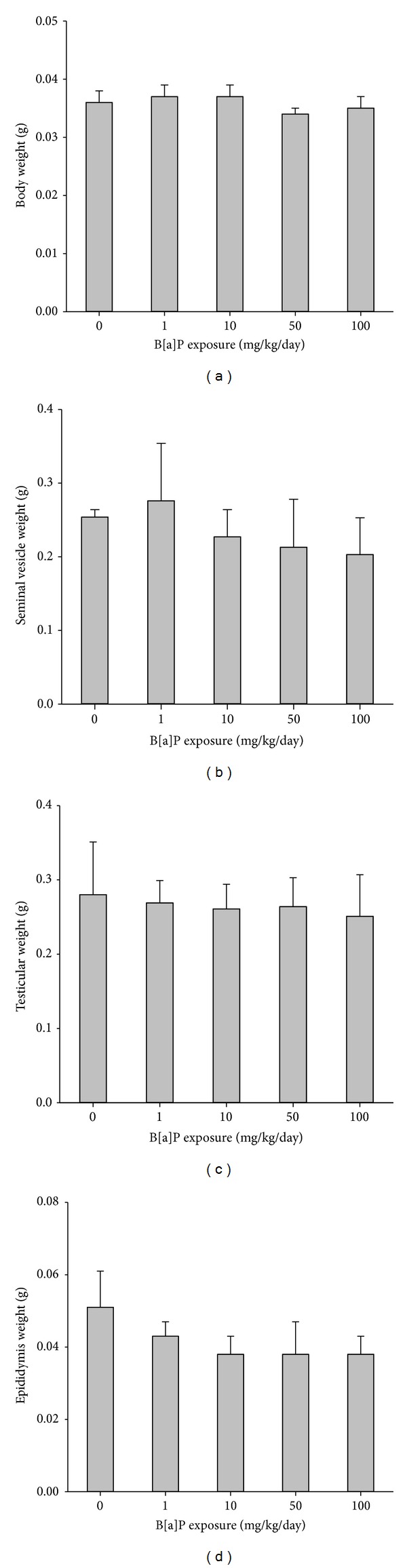
Body weight and reproductive organ weight of mice exposed to B[a]P for 30 days versus the control: (a) body weight, (b) testicular weight, (c) seminal vesicle, and (d) cauda and caput epididymis. All values are means ± SD. *Significantly different than the control (*P* < 0.05).

**Figure 2:**
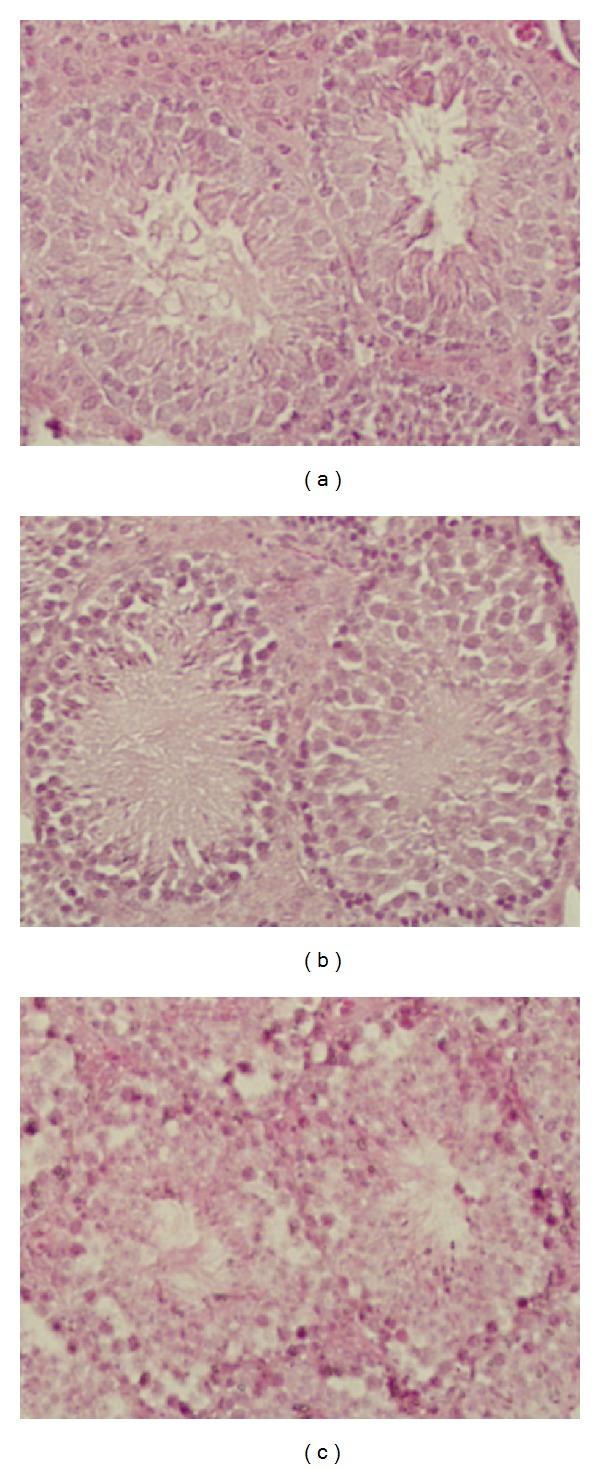
Cross section of seminiferous tubules of adult mice exposed to B[a]P at 50 mg/kg/day and 100 mg/kg/day for 30 days and the control. (a) Control, 100x showing seminiferous tubule integrity, organized seminiferous epithelium, normal luminal space, and numbers of maturing spermatozoa, (b) 50 mg/kg/day, 100x, seminiferous tubule integrity beginning to decline, seminiferous epithelium although still highly organized, showing a loss of integrity among the Sertoli cells, and (c) 100 mg/kg/day, reduced width of adluminal compartment of the seminiferous tubules, lost integrity of cellular membranes, the atrophy of seminiferous tubules, lack of spermatids and spermatozoa, and altered morphology of spermatogonia and spermatocytes.

The body weight of mice remained stable during the course of the study. The weight of testes and epididymis did gradually decrease as B[a]P doses increased (Figures 1(c) and 1(d)). However, there was no significant decrease in the weights. The cross sections of the seminiferous tubules of mice that showed that exposure to B[a]P at 100 mg/kg/day resulted in the atrophy of seminiferous tubules, reduced width of adluminal compartments of the seminiferous tubules, and altered morphology of spermatogonia and spermatocytes (Figure 2).

**Figure 3:**
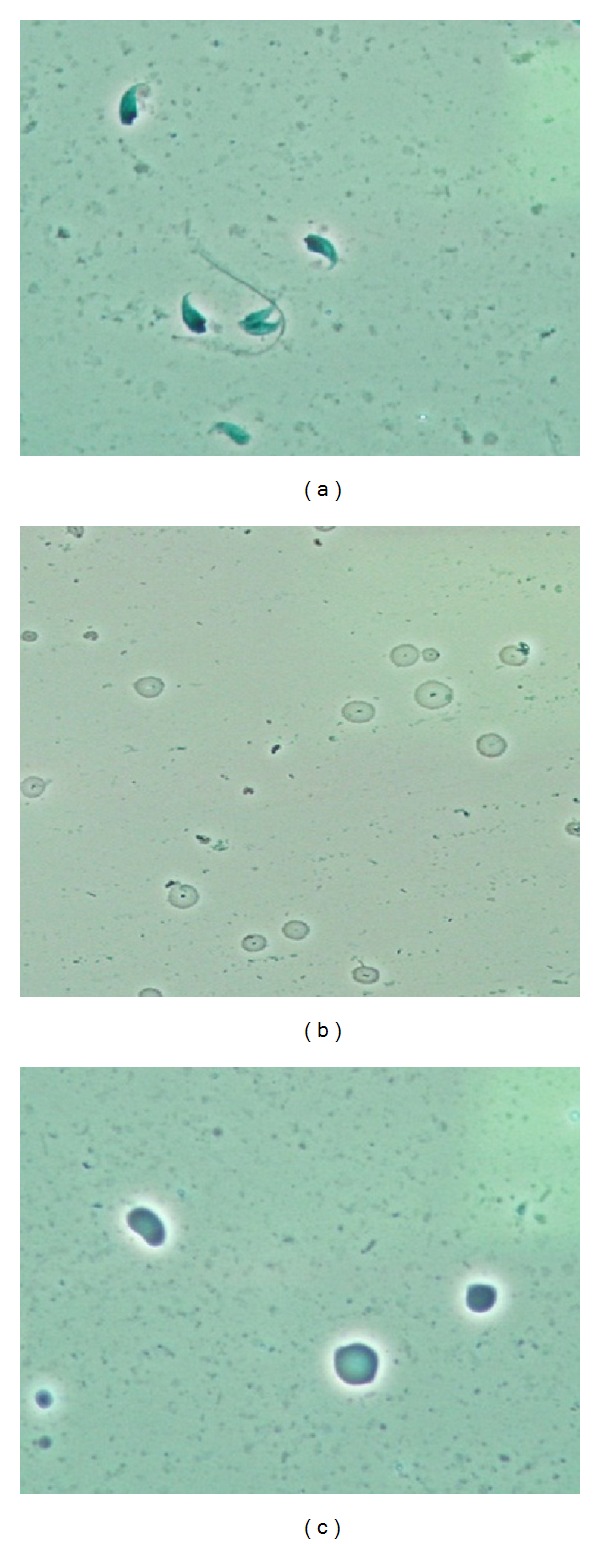
Phase contrast microscopy (oil emersion, 1000x) of spermatogenic cells: (a) elongated spermatids, (b) round spermatids, and (c) germ cells.

**Figure 4:**
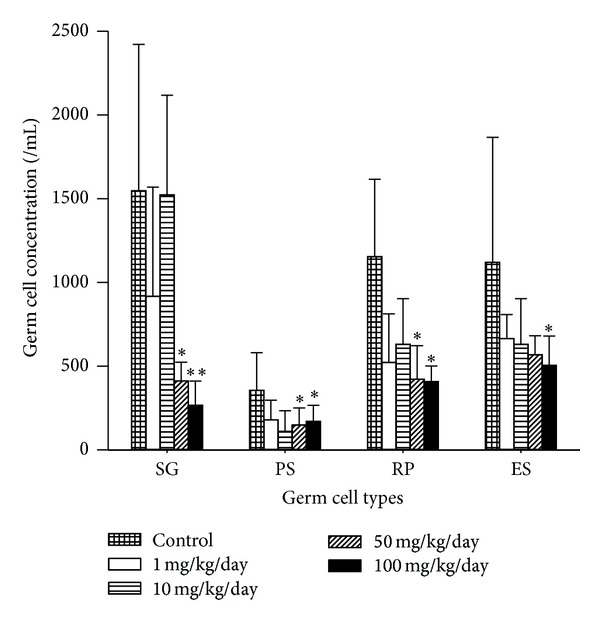
Concentrations of germ cells in mice exposed to B[a]P from 1 to 100 mg/kg/day for 30 days. SG: spermatogonia, PS: pachytene spermatocytes; ES: elongated spermatids. Germ cell values represent concentration as cells/mL. All values are means ± SD. Significance expressed as *P < 0.05, **P < 0.01.

Figure 3 shows spermatogenic cells recovered from the testes of mice exposed to B[a]P and their concentrations are shown in Figure 4 The B[a]P-treated mice yielded lower concentrations of germ cells, including spermatogonia, pachytene spermatocytes, spermatids, and elongated spermatids in the tubules, as B[a]P doses increased. However, only B[a]P at 50 mg/kg/day and 100 mg/kg/day induced a significant decrease in the concentrations of germ cells as compared to the control (*P* < 0.05).

**Figure 5:**
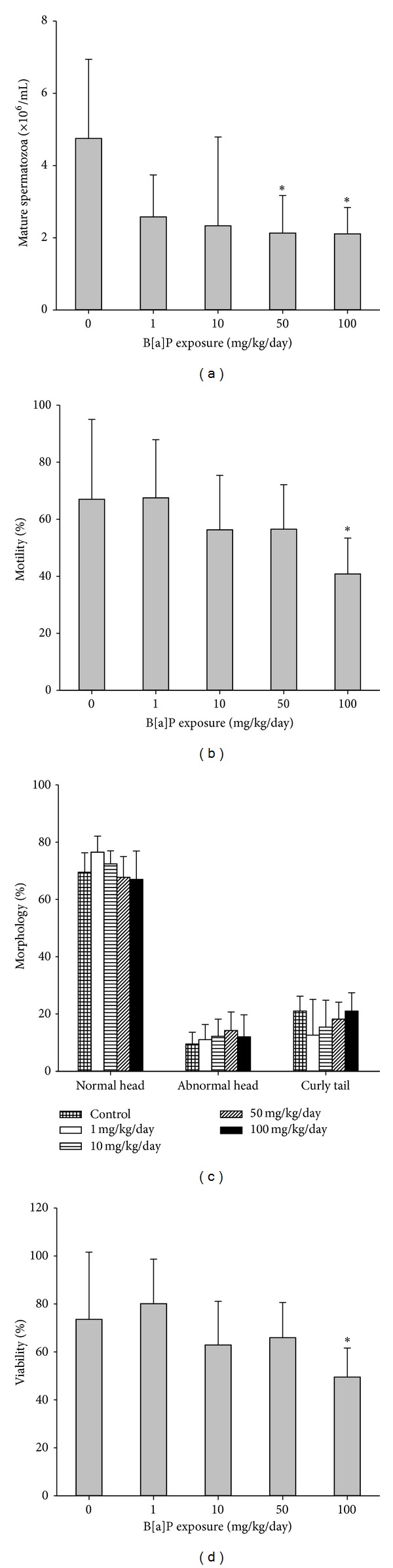
Spermatozoa quality of mice exposed to BaP and the control: (a) concentration (×10^6^/mL), (b) motility, (c) morphology, and (d) morphology, including normal head, abnormal head, and curly tail. All values are means ± SD. *P < 0.05, **P < 0.01 compared with the control.

Mice exposed to 50 and 100 mg/kg/day of B[a]P yielded significantly lower concentrations of spermatozoa than the control group (Figure 5(a)). For motility and viability, only B[a]P at 100 mg/kg/day induced a significant decrease in percentages of motility and viability as compared with the control (*P* = 0.04 and 0.04, resp.) (Figures 5(b) and 5(d)). Percentages of morphology of spermatozoa with normal head decreased but did not reach the significant level. The percentages of spermatozoa with abnormal head and curly tail remained stable with no significant changes (Figure 5(c)).

**Figure 6:**
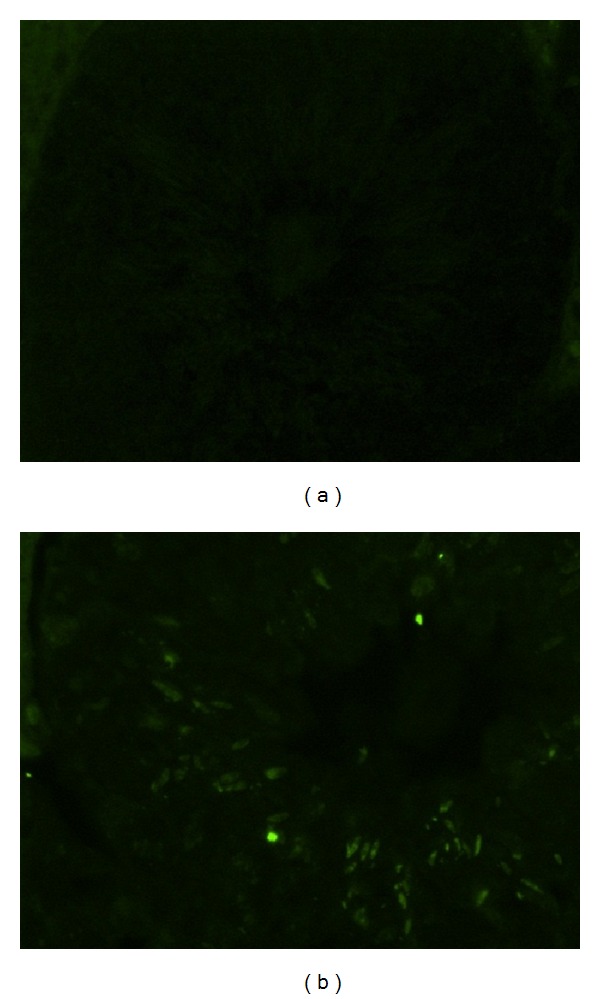
A serial cross section of a seminiferous tubule shows apoptosis in testicular germ cells detected using the TUNEL assay. (a) Control, 400x, no fluorescence indicates no apoptosis in the cells in the testicular tissue; (b) 100 mg/kg/day for 60 days, 400x, fluorescence indicates apoptosis occurring in germ cells, including spermatogonia, pachytene spermatocytes, round spermatids, and elongated spermatids.

**Figure 7:**
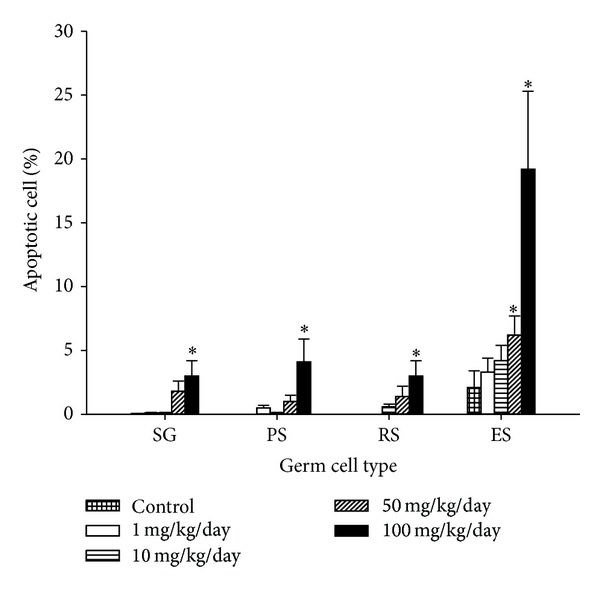
Percentage of apoptosis of germ cells from mice exposed to B[a]P with doses from 1 mg/kg/day to 100 mg/kg/day for 30 days. DNA strand breaks expressed as % positive detected using the TUNEL assay (mean ± SD and *n* = 8). *P < 0.05 versus the control.

**Figure 8:**
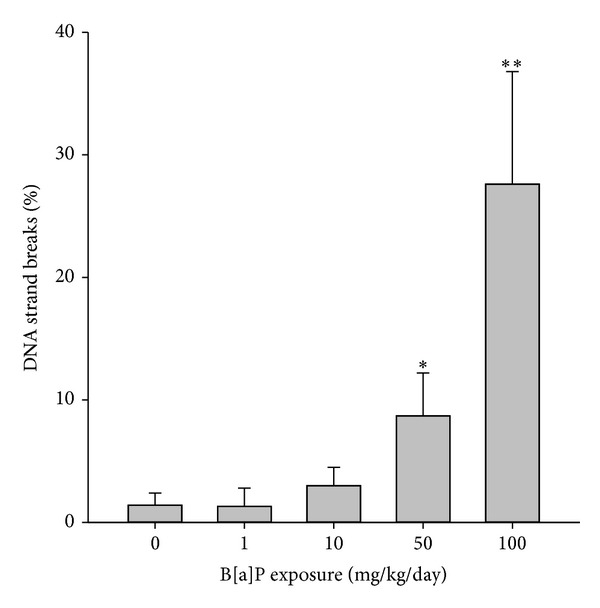
Apoptosis of spermatozoa from mice exposed to B[a]P with doses from 1 to 100 mg/kg/day for 30 days. Apoptosis was expressed as % positive detected using the TUNEL assay (mean ± SD and *n* = 8). Significance expressed as *P < 0.05, **P < 0.01.

A cross section of a seminiferous tubule of mice exposed to B[a]P at 100 mg/kg/day showed apoptosis in germ cells as detected using the TUNEL assay as compared with the control (Figure 6). Spermatogonia, pachytene spermatocytes, and round spermatids from mice exposed to ≤10 mg/kg/day had less than 0.6% of cells with apoptosis (Figure 7). The apoptosis percentages of those cells started increasing as B[a]P reached 50 mg/kg/day and 100 mg/kg/day. At 100 mg/kg/day, spermatogonia, pachytene spermatocytes, and spermatocytes had significant percentages of apoptosis (3.0%, 4.1%, and 3.0%, resp.) as compared with the control groups (0.05%, 0%, and 0%, resp.) (*P* < 0.05). Elongated spermatids were the type of germ cells with the highest percentages of cells with apoptosis. The percentages of elongated spermatids from mice exposed to 1, 10, and 50 mg/kg/day were not significantly higher than those in spermatogonia, pachytene spermatocytes, and round spermatids. At 100 mg/kg/day of B[a]P, elongated spermatids, however, had a significant increase in the percentage of apoptosis (19.2%) as compared with the control (2.1%) (*P* = 0.01). Mature spermatozoa with apoptosis had a similar pattern as elongated spermatids, whose apoptotic cells increased at 10 mg/kg/day of B[a]P and reached a significant level of increase at 50 mg/kg/day and 100 mg/kg/day (8.7% and 27.6%, resp.) as compared with the control groups (1.4%) (*P* = 0.04 and 0.009, resp.) (Figure 8).

## 4. Discussion

The current study used rodents for the experimental paradigm to provide knowledge about the influence of exposure to B[a]P on germ cells during spermatogenesis. Each type of germ cell represents steps of the spermatogenic process involving mitotic cell division, meiosis, and the process of spermiogenesis. Exposure to B[a]P, particularly at 50 mg/kg/day and 100 mg/kg/day for 30 days, induced a significant decrease in the levels of germ cells, including spermatogonia, pachytene spermatocytes, round spermatids, and elongated spermatids. However, humans do not normally encounter environmental exposure at such high doses. B[a]P could have affected the proliferation of spermatogonia and pachytene spermatocytes in the early spermatogenesis. Such effect led to the lower concentrations of round spermatids and elongated spermatids in the later spermatogenesis. The percentages of the spermatids remained the same as those of spermatozoa. Our results suggest that exposure to B[a]P did not significantly affect remodeling of spermatids during spermiogenesis, which produces the mature streamlined spermatid form.

Histological assessment revealed that B[a]P exposure at 50 mg/kg/day and 100 mg/kg/day for 30 days had a significant impact on the seminiferous tubules: reduced width of the adluminal of the seminiferous tubules and the size of the Sertoli cell basal membrane, lost integrity of cellular membranes and the atrophy of seminiferous tubules. That ran parallel with a reduction in the weight and size of testes of mice exposed to B[a]P at 100 mg/kg/day. The basal compartment of seminiferous tubules is occupied by spermatogonia and early spermatocytes and forms a niche for the developing spermatogonia [10]. The proliferation and differentiation of spermatogonia up to meiosis occurred within the basal compartment of the seminiferous tubules. A reduction in the basal membrane of Sertoli cells may impact spermatogonial proliferation and survival. Also, BaP could cause cellular changes and affect cell signaling pathways of Sertoli cells [11], which involve regulation of growth and differentiation of spermatogenic cells.

Another possible mechanism related to the reduction of germ cells could be associated with the destruction of testosterone production in Leygid cells. In mammalian male reproduction, Leygid cells are the major source of testosterone, which is a critical hormone for the initiation and maintenance of spermatogenesis. B[a]P metabolites have been detected upon accumulation in the male reproductive system, especially in the testis and epididymis of exposed animals [12]. Accumulation of the metabolites of B[a]P could reduce the number of Leydig cells via apoptosis [13]. That may lead to altering intratesticular testosterone production in Leydig cells, which causes testicular atrophy and an accompanying decrease in the number of germ cells [5, 14]. Based on the histological assessment, Leydig cells appeared to have increased DNA strand breaks (data not shown). Testosterone levels from mice exposed to B[a]P at 50 mg/kg/day and 100 mg/kg/day decreased, which was in line with increased percentages of apoptosis in spermatogonia and pachytene spermatocytes and decreased levels of the germ cells.

B[a]P promotes apoptotic cell death of four types of germ cells. With doses of B[a]P at 1 mg/kg/day and 10 mg/kg/day, the apoptotic responses of spermatogonia, pachytene spermatocytes, and round spermatids remained stable, while elongated spermatids with apoptotic responses increased. Although the percentages of apoptotic elongated spermatids were not significant higher than those in other germ cells, the elongated spermatids from mice exposed to 100 mg/kg/day had a more significant increase in apoptosis than other germ cells. During the normal spermatogenesis cycle, elongated spermatids have been known to rarely undergo apoptosis. Our results suggest that at the B[a]P doses for 30-day exposure, spermatogonia and spermatocytes with B[a]P-induced DNA damage underwent apoptotic cell death rather than repair. Then, during the course of exposure, DNA damage was passed on and accumulated in elongated spermatids. Similar results were observed in Van Loon's study, showing increased DNA breaks in the elongated spermatids after radiation exposure. The increased DNA damage might be due to a cumulative effect in the absence of repair [15]. The repair mechanisms, for example, nucleotide excision repair [16, 17], of spermatogonia seemingly failed to overcome the insults from B[a]P exposure and led to a reduction in the proliferation of pachytene spermatocytes. In addition, the high dose of 100 mg/kg/day could damage the blood-testis barrier (BTB) and directly insult the elongated spermatids.

Germ cells during spermatogenesis were susceptible to being insulted by 100 mg/kg/day of B[a]P exposure for 30 days. In the testis, junctional proteins of the Sertoli cells participated in forming BTB, which divides the seminiferous epithelium into basal and adluminal compartments. Increased levels of B[a]P-DNA adducts were detected in germ cells of mice exposed to 50 mg/kg/day and 100 mg/kg/day (data not shown). Our results suggest that B[a]P at 50 mg/kg/day and 100 mg/kg/day could compromise the function of BTB for entry into germ cells, while directly reaching germ cells in the spermatogonia and early spermatocytes. B[a]P can undergo a futile redox cycle in the presence of NADPH to generate ROS, which could directly react with germ cells and cause damage to their DNA [18].

Spermatozoa production in the testis is a regulated balance between germ cell proliferation and germ cell loss. Mice exposed to B[a]P experienced an increase in germ cell loss and a decrease in their proliferation. The reduction in germ cell levels appeared to be correlated with depletion of spermatozoa levels in the epididymis and vas deferens. Also, the percentage of apoptosis in germ cells was positively correlated with DNA damage in spermatozoa. Low concentrations of spermatozoa with poor morphology were more likely to show high levels of TUNEL positivity. Our data simply reflect that B[a]P could decrease the proliferation of germ cells that eventually leads to alteration in spermatozoa production and motility in the later stages of spermatogenesis (Figure 5). We observed that the apoptosis of germ cells appeared to be associated with the depletion of spermatozoa concentrations in the epididymis and vas deferens.

Our present study demonstrated that mice exposed to B[a]P at or greater than 50 mg/kg/day for 30 days result in pathological changes such as decreasing spermatogonial cell proliferation and modulating germ cell apoptosis during spermatocytogenesis, the early stage of spermatogenesis. These exposed mice had defects in spermatozoa production, motility, and morphology. The results suggest that B[a]P affects the apoptotic process during the early stage of spermatogenesis and leads to decreased spermatozoa quality during sperm maturation.

## 5. Conclusion

Exposure to B[a]P at higher than 50 mg/kg/day for 30 days influenced germ cell quality by decreasing their concentrations and increasing apoptotic response. B[a]P can impact germ cells during spermatogenesis, which could lead to decreased proliferation of spermatids and mature spermatozoa.

## References

[1] Xue W, Warshawsky D (2005). Metabolic activation of polycyclic and heterocyclic aromatic hydrocarbons and DNA damage: a review. *Toxicology and Applied Pharmacology*.

[2] Shultz CA, Quinn AM, Park J-H (2011). Specificity of human aldo-keto reductases, NAD(P)H: quinone oxidoreductase, and carbonyl reductases to redox-cycle polycyclic aromatic hydrocarbon diones and 4-hydroxyequilenin-o-quinone. *Chemical Research in Toxicology*.

[3] Mohamed E-SA, Song W-H, Oh S-A (2010). The transgenerational impact of benzo(a)pyrene on murine male fertility. *Human Reproduction*.

[4] Ramesh A, Walker SA, Hood DB, Guillén MD, Schneider K, Weyand EH (2004). Bioavailability and risk assessment of orally ingested polycyclic aromatic hydrocarbons. *International Journal of Toxicology*.

[5] Chung J-Y, Kim Y-J, Kim JY (2011). Benzo[a]pyrene reduces testosterone production in rat Leydig cells via a direct disturbance of testicular steroidogenic machinery. *Environmental health perspectives*.

[6] Poccia D (1986). Remodeling of nucleoproteins during gametogenesis, fertilization, and early development. *International Review of Cytology*.

[7] Romrell LJ, Bellve AR, Fawcett DW (1976). Separation of mouse spermatogenic cells by sedimentation velocity; a morphological characterization. *Developmental Biology*.

[8] Bellve AR (1993). Purification, culture, and fractionation of spermatogenic cells. *Methods in Enzymology*.

[9] Holstein A-F, Schulze W, Davidoff M (2003). Understanding spermatogenesis is a prerequisite for treatment. *Reproductive Biology and Endocrinology*.

[10] Ogawa T, Ohmura M, Ohbo K (2005). The niche for spermatogonial stem cells in the mammalian testis. *International Journal of Hematology*.

[11] Raychoudhury SS, Kubinski D (2003). Polycyclic aromatic hydrocarbon-induced cytotoxicity in cultured rat Sertoli cells involves differential apoptotic response. *Environmental health perspectives*.

[12] Ramesh A, Inyang F, Hood DB, Archibong AE, Knuckles ME, Nyanda AM (2001). Metabolism, bioavailability, and toxicokinetics of Benzo(*α*)pyrene in F-344 rats following oral administration. *Experimental and Toxicologic Pathology*.

[13] Inyang F, Ramesh A, Kopsombut P (2003). Disruption of testicular steroidogenesis and epididymal function by inhaled benzo(a)pyrene. *Reproductive Toxicology*.

[14] Awoniyi CA, Santulli R, Chandrashekar V, Schanbacher BD, Zirkin BR (1989). Quantitative restoration of advanced spermatogenic cells in adult male rats made azoospermic by active immunization against luteinizing hormone or gonadotropin-releasing hormone. *Endocrinology*.

[15] Van Loon AAWM, Sonneveld E, Hoogerbrugge J (1993). Induction and repair of DNA single-strand breaks and DNA base damage at different cellular stages of spermatogenesis of the hamster upon in vitro exposure to ionizing radiation. *Mutation Research*.

[16] Jansen J, Olsen AK, Wiger R (2001). Nucleotide excision repair in rat male germ cells: low level of repair in intact cells contrasts with high dual incision activity in vitro. *Nucleic Acids Research*.

[17] Olsen A-K, Lindeman B, Wiger R, Duale N, Brunborg G (2005). How do male germ cells handle DNA damage?. *Toxicology and Applied Pharmacology*.

[18] Abedin Z, Louis-Juste M, Stangl M, Field J (2013). The role of base excision repair genes OGG1, APN1 and APN2 in benzo[a]pyrene-7, 8-dione induced p53 mutagenesis. *Mutation Research*.

